# Dr. M’Pheeters’ Case of Chronic Diarrhœa, Treated by the Internal Use of the Nitrate of Silver

**Published:** 1842-04-23

**Authors:** W. M. McPheeters

**Affiliations:** Physician to the St. Louis Dispensary; St. Louis


*Case of Chronic Diarrhœa, treated by the internal use of the Nitrate of Silver.*

By W. M. McPheeters, Physician to the St. Louis Dispensary.

In the 51st number of the last volume of the Examiner, I saw a notice of "The internal use of nitrate of silver in inflammation of the intestines, by Dr. Macgreggor, of Dublin." About that time the following case came under my observation, in which I instituted the treatment recommended by Dr. Macgreggor, so far as the use of the nitrate of silver is concerned—which if you think of sufficient interest, you can insert in your valuable journal.

George G. æt. 40 years—for some time captain of the Philadelphia watch—of large frame and robust habits. Three years ago, while in New Orleans was attacked with diarrhœa; from which he has suffered ever since, averaging from nine to twelve stools a day; sometimes loosing all control over the sphincter, so that his discharges would pass off involuntarily. Has been treated by different physicians, as well as by quacks and empirics, but without material benefit.

January 10th, 1842, saw him for the first time in the following state:—Emaciation great, so weak as not to be able to stand alone, countenance anxious and care-worn, pulse feeble and a little excited. Bowels open from five to seven times a day, attended with bearing down pains—stools copious and frothy, of a yellow colour with some mucus. No blood; has never discovered blood in stool, at any time. No pain on pressure being made upon any part of the abdomen. Urine scanty, has been so throughout the disease, sometimes not more than an ounce during the day.

I ordered him a pill consisting of a grain of the nitrate of silver, a quarter of a grain of the watery extract of opium, and a grain and a half of the extract of gentian, three times a day. Diet light and farinaceous, with mucilaginous drinks. Ptyalism from the former use of mercury.

Jan. 12. Has had no stool since yesterday—feels more comfortable than he has for months. Treatment continued.

13. Had two stools since visit of yesterday, the first attended with considerable pain. Discharge of a tolerable consistency, without mucus.

15. Has had six stools since last visit, thin and watery with small portions of light colored fecal matter suspended. Complains of a dizziness on rising, and a glaring of the eyes. Pills discontinued. ℞. Salep powders, one three times a day, boiled in a pint of milk.

16. In the last 24 hours has had two stools—feels somewhat better, dizziness slight; appetite and thirst morbidly great.

17. Had three stools since yesterday, unattended with pain. Feels stronger and better than for several days past. Dizziness disappeared.

19. In the last 48 hours had five stools, unattended with pain and much less copious than formerly. Appetite and thirst more moderate.

23. Two days ago took cold from exposure, since which has been much worse. In the last 48 hours has had twelve or fourteen stools. ℞. Half a grain of nitrate of silver, with one-fourth of a grain of watery extract of opium, and a grain and a half of extract of gentian, three times a day.

24. Had no stool since last visit, feels better in every respect, strength improved. Powder and pills both continued.

28. Has continued to improve in strength since last visit; bowels open from three to four times in 24 hours.

Feb. 3. No material change—swelling in the feet and ankles. ℞. Mist. oleagin. ℥ss. ter in die—bandages to legs. Pills discontinued.

10. Bowels still open from four to six times in 24 hours; feet about the same. Oil mixture discontinued, ordered starch injections with tr. opii. gtt. xv. ter in die.

18. Decidedly improved since last note. For several days past has had but one stool per day, of natural consistency. Swelling of legs diminishing; ptyalism improved.

March 4. Has continued to improve daily since last visit. Diarrhœa checked; general health and strength greatly increased. Powders continued with an occasional injection.

April 5. Patient entirely recovered, and returned to occupation.

In this case, notwithstanding the long continuance of the disease, there seemed to be no appreciable lesion of the intestines. Whether the patient derived any benefit from the use of the nitrate of silver, or not, is doubtful. The advantage in the salep* powders is that with them you can control the diet of the patient with great certainty.

*St. Louis*, 9th April, 1842.

*Of salep, gum tragacanth, sago, each four ounces, of cochineal half a drachm, and of prepared chalk, an ounce.

